# Bain's Psychology
**The Senses and the Intellect*. By Alexander Bain, A.M. London. 1855.*The Emotions and the Will*. By Alexander Bain, A.M., Examiner in Logic and Moral Philosophy in the University of London. London. 1859.


**Published:** 1860-04-01

**Authors:** 


### Art. VI.—BAIN'S PSYCHOLOGY.*

WITHOUT committing ourselves to an unqualified endorsement of all Mr. Bain's detailed opinions as to the phenomena and laws of mental action, we feel no hesitation in expressing our conviction, that no single contribution has ever been made in our country to the science of mind, that has done so much to render it an accurate science as these two volumes are likely to do. This arises in part from the method adopted, and in part from the full and exhaustive manner in which this method is worked out. Mr. Bain has entirely forsaken the time-honoured *a priori* plan of investigation, which is in fact no plan at all, but chiefly a series of assumptions, and circular reasoning upon these; and has treated mental phenomena as objects of true inductive analysis; taking them singly and in combination as indicating so much value in the general result; and thereby laying a stable foundation for a system of rational *descriptive* psychology. In accomplishing this, Mr. Bain evinces profound and extensive acquaintance with all the sciences which bear directly upon mental developments, especially with physiology in most of its departments; he has also the boldness to accept as legitimate objects for reasoning the abnormal as well as the normal phases of thought, action, and feeling. We may, perhaps, question whether some of our mental states are not still too complex and too imperfectly known to admit of ultimate analysis, so that the elements can be distinctly traced and classified; and whether in attempting this, Mr. Bain has not overstrained some of his theo-

* *The Senses and the Intellect.* By Alexander Bain, A.M. London. 1855.
*The Emotions and the Will.* By Alexander Bain, A.M., Examiner in Logic and Moral Philosophy in the University of London. London. 1859.

ries, particularly, for instance, when treating of Belief, Free-will, Emotion, &c.; this we may question or suspect, but granting all this or more, few will deny after carefully reading these interesting and elaborate volumes, that the author has rendered a service to our science which may now be partially recognised, but which can only be fully appreciated perhaps after the labours of many others have built an enduring system upon his foundation.

Having premised thus far upon the general character of the work, we believe that instead of further defending our author's method and indicating his precise place amongst the *schoolmen* (a work for which we imagine he would be little recognisant), it will be more acceptable to our readers to place before them a concise analysis of the plan, with a somewhat more detailed account of such parts of the theory (and these are many and important) as are entirely new.

Mr. Bain recognises in the outset three attributes or capacities of mind:—

1. It has Feeling, in which is included what is commonly called Sensation and Emotion.
2. It can Act, according to Feeling.
3. It can Think.

On this classification we may remark that Feeling, Emotion, and Consciousness are terms used "to express one and the same attribute of mind."* In the second attribute so simply stated, we trace one of the author's most defined views, that voluntary action results exclusively from feeling; a principle which afterwards materially influences his views upon volition; the will appearing but as the result of the balance of motives.

An important consideration is broadly stated immediately after this classification:—

"Consciousness is inseparable from the first of these capacities, but not, as it appears to me, from the second or the third. True, our actions and thoughts are usually conscious, that is, as known to us by an inward perception; but the consciousness of an act is manifestly not the act, and although the assertion is less obvious, I believe that the consciousness of a thought is distinct from the thought. To flee on the appearance of danger is one thing, and to be conscious that we apprehend danger is another."†

We believe that all this is true, but we should go further *if* thus far. For by whatever process of reasoning Thought can be represented as unconscious, by the same can Emotion be so viewed.

* See p. 1, Introduction, vol. i. In all these references the treatise on the *Senses and the Intellect* is considered as the first volume, and that on the *Emotions and the Will* as the second.

† *Loc. cit.*

And if Action may be unconscious, and at the same time the result of Feeling, as by the terms of the second definition it is supposed to be, the motor cause we should infer should be (*possibly* at least) as unconscious as the resultant act. But in the present stage of the investigation it would be premature to enter more fully into this question.

There is nothing requiring specific notice in the preliminary general notions given of Feeling or Action; those concerning Thought are worthy of quotation:—

"The first fact implied in it (*i.e.*, Thought or Intelligence) is *discrimination*, with sense of agreement or difference, as when of two things taken into the mouth the animal prefers one to the other. . . . To go back upon a former experience as preferable to the present is to act upon an idea, a thought; whenever this is clearly manifested we see an intelligent being.

"Another fact of intelligence, also exhibited by the lower orders of creatures, is the power of associating ends with means or instruments, so as to dictate intermediate actions. . . . . .

"These two facts, discriminating with preference, and the performance of intermediate actions to attain an end, are the most universal aspects of intelligence, inasmuch as they pervade the whole of the animal creation."*

Perhaps this is an assumption rather too general and hasty. It can scarcely be doubted that there are large tribes of creatures clearly recognisable as of animal nature, in which we certainly have no *evidence* of *discrimination* in the sense here implied, though that it *may be* there is not impossible. In many of the same creatures there is the same lack of any *proof* that ends are intelligently associated with means. The author continues thus:—

"In the higher regions of mind, the attribute of thinking implies the storing up, reviving, and combining anew all the impressions constituting what we call knowledge, and principally derived from the outer world acting on the senses. It is this wider range of intellectual operations displayed by the human mind, that gives scope for exposition in a work like the present.

"Although in the animal constitution Thought is coupled and conjoined with Feeling and Volition, it does not necessarily follow that Intelligence is a necessary part of either the one or the other. I have a difficulty in supposing Volition to operate in the entire absence of an intellectual nature, nevertheless I cannot help looking upon the intellect as a distinct endowment, following laws of its own, being sometimes well developed and sometimes feeble, without regard to the force or degree of the two other attributes."†

In proceeding to consider the phenomena of mind, Mr. Bain enters briefly upon the proofs (sufficiently familiar to physiolo-

* Vol. i. Introduction, p. 6. † *Ibid.*

gists) that the brain and nervous centres are the organs to which its manifestations are due, or with which they are connected. A concise account follows of the nervous centres and the organs of sense and locomotion. Throughout this work, mind is treated of as closely connected, inseparably connected, with the actions of a material organism; not, so far as we can trace, from any leaning towards what is generally called materialism, but simply for convenience, inasmuch as *all* our experience of mind is derived through and from the observation of material changes by means of our material organs; and in a system so purely inductive and descriptive as the present it is absolutely necessary to state and recognise that, so far as we can know, where mind acts, there matter is in change; and where these changes are to be observed, it must be through material organs of sense. We believe that the question of the materiality or immateriality of mind is not once alluded to, nor indeed the *nature* of mind at all; it is with phenomena only that this work deals.

One of the views most distinctive of, and most extensively affecting, the entire theory set forth in these volumes, is connected with "Spontaneous Activity." So far from holding the popularly received doctrine that sensation originates motion, Mr. Bain upholds "that movement precedes sensation, and is at the outset independent of any stimulus from without; and that action is a more intimate and inseparable property of our constitution than any of our sensations, and in fact enters as a component part into every one of the senses, giving them the character of compounds, while itself is a simple and elementary property."* This doctrine is inextricably interwoven with the entire theory of volition; in the mode in which it is brought forward it has the aspect of novelty; the acquisition of voluntary acts is placed by it upon an entirely new basis; on these grounds we shall enter at more detail into our author's line of argument.

The first step is to prove the "existence of a class of movements and actions anterior to, and independent of, the sensations of the senses." These movements arise, according to this view, from the central stimulus of the nervous system, and are "the spontaneous discharge of the active energy of the nerve centres."†

The proofs adduced are as follow:—

"1. The tonicity of muscles; not amounting to motion, but the tension implying a lower degree of similar activity.

"2. The permanent closure of the sphincter muscles, not accounted for either by impressions from within or without the body, or by the muscles' own contractility, inasmuch as the destruction of certain nerve centres relaxes these muscles.

"3. The action of the involuntary muscles."

* Vol. i. p. 67. † Vol. i. p. 83.

On which argument, however, the author judiciously refrains from laying much stress.

"4. In awakening from sleep, movement precedes sensation. If light were essential to the movements concerned in vision, it would be impossible to open the eyes. The act of awakening from sleep can hardly be considered in any other view than as the reviving of the activity by a rush of nervous power to the muscles, FOLLOWED by the exposure of the senses to the influences of the outer world. I know of no circumstance that would go to show that sensation is the antecedent fact, in the case when the individual wakes of his own accord. The first symptom of awakening that presents itself is a general commotion of the frame, a number of spontaneous movements—the stretching of the limbs, the opening of the eyes, the expansion of the features —to all which succeeds the revival of the sensibility to outward things. Mysterious as the nature of sleep is in the present state of our knowledge, we are not precluded from remarking so notable a circumstance as the priority of action to sensibility, at the moment of wakening.

"But if this be a fact, we seem to prove beyond a doubt that the renewed action must originate with the nerve centres themselves. The first gestures must be stimulated from within, by a power lodged in the grey masses of the brain; afterwards they are linked with the gestures and movements suggested by sense, and revived by intelligence and will. . . . We are at liberty to suppose that the nourished condition of the nerve centres, consequent on the night's repose, is the cause of that burst of spontaneous exertion which marks the movement of awakening. The antecedent of the activity in this case is therefore more physical than mental, and this must be the case with spontaneous energy in general."*

Before passing on to another order of proofs adduced by the author, we would make one or two remarks on those already given. We are inclined to *believe* in the spontaneous activity here indicated, inasmuch as we can see no reason why nutrition and rest should not induce a state of polarity in the nervous centres, which finds its restoration to equilibrium by this sort of discharge; we can, on the contrary, see many cogent reasons why it should be so. But the fact must not be overlooked that these arguments do not *prove* the case,—that they are indeed perfectly explicable on the converse hypothesis. We need not enter into the physiological refutation of the first three sections; the fourth claims a few remarks. We doubt extremely whether, save in very exceptional instances, motion does precede sensation in the act of awaking. (1) We are all conscious, very frequently, of a more or less prolonged interval between our first waking idea (*i. e.*, sensation), and our first motion or action; when existence appears to be nothing but a pure sensation. (2) The active phenomena of awakening, as above described, agree accurately with

* Vol. i. pp. 75–6.

those almost invariably observed when the waking state is brought about by an external stimulus or influence upon the senses. (3) We awaken in a precisely similar manner, when we do so in obedience to some idea (sensation) conveyed in a dream. (4) Not unfrequently, when we awake suddenly with a start, and motion *appears* to be the first link of the waking chain, we remember, some time afterwards, that we were dreaming, and received a sudden shock, ideally, in the dream which caused the start. (5) The opening of the eyes, previous to the stimulus of light, can count for nothing as a proof, because it would occur equally in the dark in obedience to any sudden call, or impulse, as part of the co-ordinate movements. (6) It is quite impossible to prove that an *organic* sensation does not pre-exist in all cases of awakening, even supposing that no dream, or conscious interval or idea should be present. Thus far, therefore, although inclined to agree with the theory, we are in doubt as to the cogency of the arguments adduced in proof. We will now, however, examine those derived from other considerations.

5. The early movements of infancy are supposed by our author to be in great part due to the spontaneous activity of the nervous centres. Some part of these movements may be attributable to the stimulus of sensation, to the sights, sounds, and movements of outward things;—some part again to emotion or sensation generated within the body, or to states of consciousness growing out of the brain and the bodily processes generally, "as when internal pain gives rise to paroxysms, or high health to the lively movements of mere animal spirits;" but as these appear to be actions and gesticulations which show no connexion with sight or sound, or other influence of the external world, and also that have no particular motional character either of pleasure or pain, it appears that "we can ascribe them to nothing but the mere abundance and exuberance of self-acting muscular and cerebral energy, which will rise and fall with the vigour and nourishment of the general system."*

The activity of young animals in general, and especially of such animals as are remarkable for their active endowments, as insects, seems to us to be the strongest argument brought forward to prove the spontaneity of muscular action:—

"When the kitten plays with a worsted ball, we always attribute the overflowing fulness of moving energy to the creature's own inward stimulus, to which the ball merely serves for a pretext. So an active young hound, refreshed by sleep or rested by confinement, pants for being let loose, not because of anything that attracts his view, or kindles up his ear, but because a rush of activity courses through his members, rendering him uneasy till the confined energy has found vent-

* Vol. i. p. 77.

in a chase or a run. We are at no loss to distinguish this kind of activity from that awakened by sensation or emotion, and the distinction is accordingly recognised in the modes of interpreting the movements and feelings of animals. When a rider speaks of his horse as 'fresh,' he implies that the natural activity is undischarged, and pressing for vent; the excitement caused by mixing in a chase or in a battle, is a totally different thing from the spontaneous vehemence of a full-fed and under-worked animal."*

In like manner it would appear as though the activity of early human life ought to be attributed in great measure, neither to sensation nor emotion, but to "freshness"—to a current of undischarged activity. High health, natural vigour, and spontaneous outpouring appear frequently to be the only obvious antecedents of ebullient activity. "The very necessity of bodily exercise felt by every one, and most of all by the young, is a proof of the existence of a fund of energy that comes round with the day, and presses to be discharged."

The remaining arguments for muscular spontaneity are more complex, and *pro tanto* more open to objection. They are founded upon extreme activity as dependent upon excitement,—upon the fact that sensibility and activity are not proportionate one to the other (an argument open to much discussion); and upon the consideration that without this spontaneity, volition, or "activity guided to ends," would be impossible. We shall see shortly how this last position is developed; and that we may do this and keep up the connexion, we will pass over without comment the other arguments.

In the chapter on the "Instinctive Germ of Volition" (p. 289) we find it stated that, "this fact of spontaneous activity I look upon as an essential prelude to voluntary power, making, indeed, one of the terms or elements of volition; in other words, volition is a compound, made up of this and something else." What is it then, that is superadded to spontaneous motion of limbs, body, voice, tongue, eyes, &c., to produce volition?

"If we look at this kind of (spontaneous) impulse closely, we shall see wherein its defect or insufficiency lies, namely, in the random nature of it; being dependent on the condition of the various nervous centres, the discharge is regulated by physical circumstances, and not by the ends, purposes, or uses of the animal."†

Mr. Bain's theory of the growth of volition being quite new, we shall give it in his own words, although the quotation is somewhat lengthy. "I will endeavour to indicate what seems to me to be the circumstance that leads to this remarkable union between the two great isolated facts of our nature; namely, on the one hand, feelings inciting to movement in general, but to no action

* Vol. i. p. 77. † Vol. i. p. 291.

in particular, and, on the other hand, the spontaneous movements already spoken of." Our readers will do well to note the next sentence and the italics which are our own, as directing attention to Mr. Bain's special views as to the purely accidental origin of each particular act of volition:—

"*If* at the moment of some acute pain, there should *accidentally occur* a spontaneous movement, and *if* that movement sensibly alleviates the pain, then it is that the volitional impulse belonging to the feeling will show itself. The movement *accidentally begun* through some other influence, will be sustained through this influence of the painful emotion. In the original situation of things, the acute feeling is unable of itself to bring on the precise movement that would modify the suffering; there is no primordial link between a state of suffering and a train of alleviating movements. But should the proper movement be once actually begun, and cause a felt diminution of the acute agony, the spur that belongs to states of pain would suffice to sustain this movement. . . . If the state of pain cannot awaken a dormant action, a present feeling can at least maintain a present action. This, so far as I can make out, is the original position of things in the matter of volition. . . . . An example will perhaps place this speculation in a clearer light. An infant lying in bed has the painful sensation of chilliness. This feeling produces the usual emotional display, namely, movements, and perhaps cries and tears. Besides these emotional elements, there is a latent spur of volition, but with nothing to lay hold of as yet, owing to the disconnected condition of the mental arrangements at our birth. The child's spontaneity, however, may be awake, and the pained condition will act so as to irritate the spontaneous centres, and make their central stimulus flow more copiously. In the course of a variety of spontaneous movements of arms, legs, and body, there occurs an action that brings the child in contact with the nurse lying beside it; instantly warmth is felt, and this alleviation of the painful feeling becomes immediately the stimulus to sustain the movement going on at that moment. That movement, when discovered, is kept up in preference to the others occurring in the course of the random spontaneity. . . . .

"By a process of cohesion or acquisition, which I shall afterwards dwell upon, the movement and the feeling become so linked together, that the feeling can at after times awaken the movement out of dormancy; this is the state of matters in the maturity of volition. The infant of twelve months' old can hitch nearer the side of the nurse, although no spontaneous movements to that effect happen at the moment; past repetition has established a connexion that did not exist at the beginning, whereby the feeling and action have become linked together as cause and effect. A full-grown volition is now manifested, instead of that vague incitement that could do nothing until the right movement had sprung up in the course of a series of spontaneous discharges of the central sources of power."*

* Vol. i. pp. 294–6.

The child that begins to suck when the nipple is placed between its lips, does so by virtue of a reflex action; but Mr. Bain considers that its continuing to do so, so long as the sensation of hunger is felt, and its ceasing when that sensation ceases, are truly volitional acts. The theory is thus summed up:—

"1. There is a power of spontaneous movement in the various active organs anterior to, and independent of, the feelings that such movement may give birth to; and without this, no action for an end can ever be commenced.

2. There exists consciousness, feeling, sensation, or emotion, produced from movements, from stimulants of the senses and sensitive parts, or from other causes. The physical accompaniment of this is a diffused excitement of the bodily organs constituting the outburst or expression of it, as the start from a blow.

3. There is a property of consciousness—superadded to, and by no means involved in, this diffused energy of expression—whereby a feeling can influence any present active exertion of the body, so as either to continue or abate that exertion. This is the property that links feeling to movement, thereby giving birth to volition.

The feelings that possess this power—including nearly all the pains and many states of pleasure—I have hitherto described as volitional feelings; those that are deficient in this stimulus, being principally of the pleasurable class, are the pure, unvolitional, or serene emotions."

It is a singularly novel idea, that to accident we should be indebted for all our voluntary powers. In saying this, it is with no intention of placing the theory in an absurd light; there is very much in it deserving of the most careful consideration; there is much which, if true, will throw a very different light upon many questions connected with psychology. For although we have hitherto noticed only the most elementary efforts of volition, Mr. Bain afterwards shows clearly that *all* our voluntary acts, even to the minutest detail, must necessarily have the same accidental origin. There are certain objections which strike us at the outset, which we may as well state briefly, before following our author in his further account of volition.

Unless there be some very definite proof of an accidental origin of what we have been accustomed to consider instances of special design, we should be slow to receive such an hypothesis. Very lately an ingenious work* has been published, attempting to account for all the varieties of animal and vegetable life on the principle of accidental variations of individuals, the selection of useful varieties, or such as give their possessor an advantage over others in the struggle for existence, and the propaga-

* Darwin on the *Origin of Species by means of Natural Selection.*

tion of such variations, and their accumulation, in successive generations. On examination there appears to be a total lack of any proof or even evidence of the probability of such a view; the theory treats of events as they might have been, not as they are. This theory of Mr. Bain's has reminded us strongly of the one just mentioned, in so far as he attributes all our actions to chance originally, those being selected for continuance which are of service, or pleasant, those being rejected which are useless, or unpleasing. In our author's theory there certainly is not an entire want of evidence, nor of probability in some aspects; and yet we do not feel that it fulfils the requirements of the phenomena.

In the first place, taking the earliest acts of life, we cannot say with certainty that whilst beginning to walk is a reflex act, its continuance or cessation depends upon volition. On the contrary, we should be inclined to consider both as automatic, for reasons that will readily suggest themselves to the physiologist.

Secondly, we have no evidence that a child, having performed numberless spontaneous, unconnected, and incoherent acts, does really recognise that any one is beneficial, and actually selects it for continuance, systematically rejecting the others. Perhaps what direct evidence we can gather would tend rather to controvert this idea. The child continues to kick even after it feels the required warmth; it continues to cry although this does not alleviate the pain. It never seems in the earliest days to *select* anything; the most that we can say is, that at one period or other we do observe actions appearing like means to ends, and that before this we have observed actions not having this character. Whether the one class results from the other, is as yet a question of pure theory. Certainly when we do see volition *at all marked*, it is with some obviousness of purpose. When a child first stretches out its hand towards the light, or to seize any bright object, we can scarcely conceive by what purely accidental manœuvres it can have arrived at that process. According to the theory, this should be a very late process; every muscle and every combination of muscles should have previously been over and over again thrown into action under similar circumstances, and the results marked and analysed, before the child could ascertain that it could grasp an object by reaching out the arm and hand. Except by the continuance of an act once accidentally begun, it can by the terms of this theory accomplish nothing, and can by no means know that the arm will have any more prehensile faculty than the muscles of the back. How, then, is it to arrive at any voluntary power, without going through the millions of combinations of which the numerous muscles of the body will admit? Our author would answer that all its acts are experimental, and that some time or other they succeed; it may be so, but the proof is wanting.

Another objection that we have is founded upon the idea that were the origin of voluntary actions so accidental as is here represented, seeing that the elements of action are so numerous and complex, we ought to find greater variety and uncertainty in the results than we do; for almost all children arrive shortly at nearly the same results. The full discussion of this subject, however, would involve us in too lengthy an argument for our present purpose.

The last objection that we shall at present bring forward is founded upon the early acts of young animals. When the young duck seeks the water and swims upon it, surely this is not the result of an almost interminable series of vague strivings and incoherent actions. When the calf rises and sucks the teat of its mother, it is done with a precision that is only modified by the weakness of its muscular system. The same may be said of all the earliest actions of animal life. If it be answered that these are instinctive acts, we should reply that the earliest acts of human life are certainly founded upon instinct as clearly and distinctly as those of other animals, although they are later in their development.

We may now follow our author in his further development of the origin and growth of volition. The *energy* of volition appears to be determined in great measure by that of those spontaneous movements from which it originates, and to this many elements contribute. The first is the natural vigour of the constitution. "Youth and health, the plentiful nourishment and absence of drain, the damming up of the accumulated charge by temporary restraint—are predisposing causes of a great and sudden outburst, during which the individual's active capacity is at the highest pitch. . . . . The boy let out from school, incontinently leaps over ditches, breaks down barriers, and displaces heavy bodies, and should these operations be wanted at the moment, no special or extraordinary stimulus would be needed to bring the requisite power into play."* Other influences may be briefly summed up—excitement, stimulus of pain or pleasure, emotions of fear, anger, resentment, &c.

Some interesting remarks succeed concerning the linking together of feeling and action—a great mystery in the mental constitution. Mr. Bain considers it an ultimate fact in our nature, an original property of our feelings, to prompt the active system one way or another,—the property of a painful consciousness being to stimulate action, to clutch hold of and retain any movement that alleviates the pain, whilst that of a pleasant consciousness is

* Vol. ii. p. 335.

to stimulate the continuance of an act inducing it. All this must however occur after many vain and futile attempts, and by the merest accident. "The first steps of our volitional education are a jumble of spluttering, stumbling, and all but despairing hopelessness. Instead of a clear and distinct curriculum, we have to wait upon the accidents, and improve them when they come."* Mr. Bain on one occasion (p. 352, vol. ii.) adduces an elementary proof of these positions, which we think admits of more than doubt. He says, "The spontaneous action that brings a limb into a painful contact, as when the child kicks its foot against a pin in its dress, is undoubtedly from the earliest moment of mental life arrested. Without this I see no possible commencement of voluntary power." Much observation of the manner in which children comport themselves under painful impressions would lead us to a view directly opposite to this; we believe that it is the result of long and much later experience that leads a child to cease a painful act, or to continue a pleasing one.

How far Mr. Bain carries his theory may be seen by the following extracts (See vol. ii. p. 356, *et sequent.*) :—

"I will not vouch for the truth of an assertion frequently made, that some animals, as the duckling, know water by sight before drinking it. This much is certain, that a thirsty creature having once got water into its mouth, feels a very great change of sensation, and this change for the better operates indirectly in sustaining the act, whatever it is, that administers the relief. . . . .

"Still it would be a very long period before a creature would, in ordinary circumstances, come upon a pool of water, make experiments upon its properties, and get upon the right movement for imbibing it; if this were requisite for supporting life on the first day, few land animals could live. The satisfying of the thirst at the outset is due to the mother's milk, (but where is the milk of the duck?) or the moisture of the food; and by-and-by in the course of its rambles and pokings, the young animal encounters a stream, and applies its mouth to the surface, (why not its tail? since by the hypothesis all must be accidental or spontaneous;) putting out the tongue, and executing some of those movements of tongue and jaw already associated with the contact of objects of food. The refreshing sensation that follows maintains to the point of satiety the action begun; and an effective lesson is gone through, in uniting by an enduring association the two elements thus brought into conjunction. After a very few such occasions, the contact of the cool liquid with the parched mouth brings at once into play the movements of imbibition, for which we may be assured there was no original provision, independent of successful trials confirmed by the adhesive power of the mind."

It would require very little demonstration to prove that this

* Vol. ii. p. 343.

gradual construction of the voluntary powers is in no respect accordant with the phenomena of early instincts. But we would only notice one point with reference to imbibition. There are a certain and considerable number of muscles involved in this act, from those enabling the animal to stoop (say) to the water, to the last propulsive act of the pharynx. Now as each act of each muscle must originally be accidental and tentative, and as it is absolutely necessary for the perfection of the complete act that all these muscles must contract in definite order and rhythm, we would propose it as a problem in the doctrine of chances, to determine how many millions of chances there are against any one animal ever learning to drink at all; and what a bare possibility that it might be acquired at last.

Mr. Bain, however, believes that all voluntary acts, instinctive or otherwise, are the result of experience. Animals crouch together because they *find* that it promotes warmth (see Vol. II. p. 358). They lie close one to another, and creep into holes and corners, that they may stave off the cold, or sustain the pleasure of the heat; these being "portions of the acquired experience of the animal tribes." After dwelling at some length upon the influence of the various appetites over the development of volition, the author announces the following rather startling view as to the appetite of sex:—

"The remaining appetite, sex, would constitute an opposite instance, if studied in the animal tribes. *The means of gratifying this appetite are not instinctively known*, so far as we are able to judge; and *therefore a process of groping must precede the mature faculty.* The attempt not being entered upon until the animal is in every other respect master of its movements, the difficulty is lessened to a very great degree, but for which one does not see how such an act could ever be hit upon by the generality of creatures. The remarkable intensity of the resulting feeling easily explains the persistence, when once initiation has taken place."*

No experience, so far as we have seen, tends in the slightest degree to confirm such a view as the one held by Mr. Bain. We believe in conclusion that his account of the origin, nature, and development of volition is not theoretically coherent, nor is it consistent with observed facts.

We have dwelt at length upon volition, because it presents the greatest novelty of idea, and the greatest fertility of resource in its support. The treatises on the Sensations, Emotions, and the Intellect, contain matter of the highest excellence and interest; with less that is strictly original or new; less matter for dispute; but much to excite admiration from the clear and philosophical treatment awarded to each subject in turn—from the depth and

* Vol. ii. pp. 371–2.

breadth of thought manifested throughout—and from the extent of scientific illustration brought to bear upon many of the most important and difficult problems of our nature. If Mr. Bain has not given to the world a perfect system of descriptive and analytic psychology, he has at least done that which will enable less original minds to build upon his foundation an enduring edifice; he has boldly struck out a new path in mental science, and has rendered more service to the cause than many centuries of merely speculative metaphysicians.

---

